# Practice of Cryopreservation of Cellular Starting Materials from the Asia-Pacific Region: An Industrial Perspective

**DOI:** 10.1007/s43441-025-00808-9

**Published:** 2025-06-03

**Authors:** Yun Hsiang Chang, Yuma Fujimori, Cheng Chieh Chen, Masayo Urabe, Taeko Karaki, Yusuke Izumi, Kaoru Kaneko, Kisaragi Mochiki

**Affiliations:** Janssen Pharmaceutical K.K., 3 Chome-5-2 Nishikanda, Chiyoda-ku, Tokyo, 101-0065 Japan

**Keywords:** Asia Pacific, Chimeric antigen receptor, Health care economics, Immunotherapy, Regulatory

## Abstract

Chimeric antigen receptor T-cell (CAR-T) therapy uses autologous T cells from patients to eliminate malignant targets. Cryopreservation of cellular starting materials, particularly fresh leukocytes, is an important step before production. While this promising specialized immune therapy is advancing, regulations have evolved, as specified in the US (21CFR1271) and Europe (EU Annex 1, 1394/2007). Cryopreservation is considered by this as minimal manipulation or is not considered as substantial manipulation unless there is alteration of relevant biological cell characteristics or cellular engineering. Similar consideration has been made by health authorities in Australia and South Korea. Conversely, the health authority in Japan determines if the starting material is applicable to Good Gene, Cellular, and Tissue-based Products Manufacturing Practice based on scientific data regarding the impact on product quality and safety. Whereas regulations have evolved in the US and EU, this is the first article to systemically review, from a manufacturer’s perspective, the specific regulatory positions taken towards cryopreservation in Asia-Pacific (APAC) countries, i.e. Japan, Australia and South Korea. These positions generally consider that formulation and cryopreservation should be performed in a closed system, thus protecting cellular starting materials from contaminant exposure with a low-risk approach. Local and centralized cryopreservation logistics are discussed along with optimal implementation practices. The impact of geographic access on cryopreservation logistics, as well as the importance of careful evaluation of logistical and cost aspects for successful supply of CAR-T therapies in APAC, are also discussed.

## Introduction

Cancer immunotherapy has emerged as a promising treatment option that redirects the immune system to target cancer cells. Chimeric antigen receptor (CAR)-T cell therapy, a form of personalized cancer immunotherapy, has gained attention due to its potential to cure hematologic malignancies [1, 2].

CAR-T therapeutic product manufacturing is fundamentally different from that of traditional oncologic drugs, requiring a synchronized approach from supply chain professionals, healthcare providers, and regulators [3]. Regulatory agencies have developed new requirements for cellular starting materials, i.e. leukapheresis material, such as those of the Australian Code of Good Manufacturing Practice for Human Blood and Blood Components, Human Tissues and Human Cellular Therapy Products (Australian GMPs for HCT/Ps) [4], the Japanese Ministry of Health, Labor, and Welfare (the Good Gene, Cellular, and Tissue**-**based Products Manufacturing Practice (GCTP) [5], and the Korean Ministry of Food and Drug Safety (Act on the Safety of and Support for Advanced Regenerative Medicine and Advanced Biological Products (ARMAB) [6]. These requirements emphasize the critical nature of cold chain logistics (i.e. temperature management), end-to-end chain of custody/identity, and prevention of cross-contamination of cellular starting materials. Further, personnel and processing environment aspects require consideration as it is impossible to sterilize cellular starting materials in the downstream production process. Satisfying regulatory requirements by taking a risk-based approach towards product development and manufacturing based on scientific evidence should avoid unnecessary overinterpretation of regulations, especially when guidance is unclear [7]. It is also important to consider the critical quality attributes of cellular starting materials, including those related to cryopreservation such as safety and purity [8].

This manufacturer’s perspective of the regulatory environment and logistics of cryopreservation of fresh leukocytes for CAR-T therapy in the Asia-Pacific (APAC) region discusses best practices for implementing cryopreservation logistics. We also discuss the importance of careful evaluation of logistics, cost, and quality control aspects for successful manufacturing of CAR-T therapeutic products in the APAC region.

It should be noted that the Chinese market is out of scope of this article. In China, domestic CAR-T therapies are priced significantly lower to improve patient access in a market where healthcare resources may be limited and cost sensitive. This has an impact on the market entry strategy of pharmaceutical companies in that foreign firms outside China are often required to adapt their strategies by offering patient assistance programs or collaborating with local companies to explore more sustainable pricing models rather than applying their own strategies for market entry into China.

## Implementing Cryopreservation Logistics for Autologous CAR-T Manufacturing in a Global Supply Chain

**Fig. 1:**
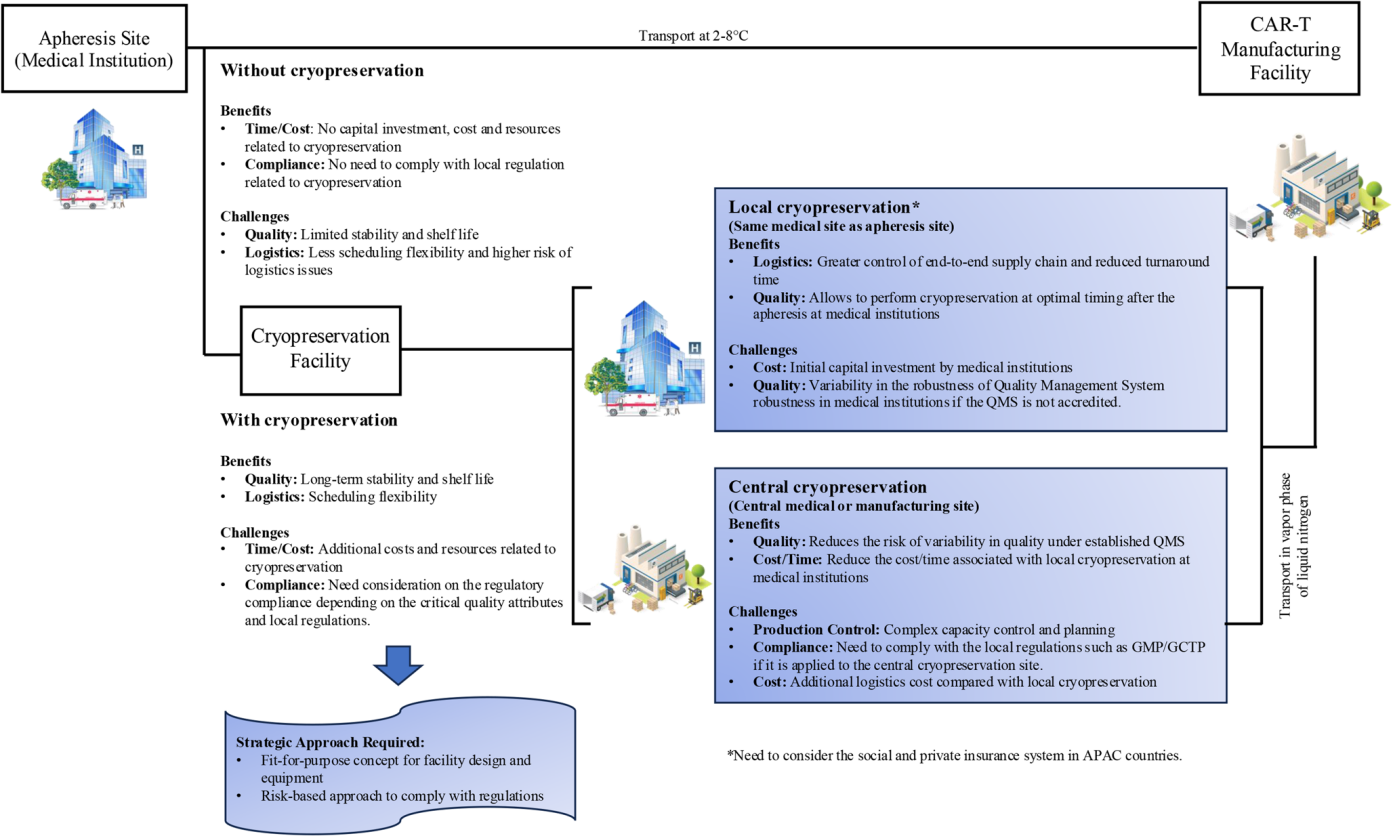
Overview of fresh versus cryopreserved apheresis and local versus central cryopreservation strategies. Abbreviations: CAR-T, Chimeric antigen receptor T-cell; GCTP, Good Gene, Cellular, and Tissue-based Products Manufacturing Practice; GMP, Good Manufacturing Practice; QMS, Quality Management System

Manufacturers wishing to create a controlled global supply chain for autologous CAR-T manufacturing face significant concerns relating to leukapheresis starting material logistics [3]. Fresh apheresis material for cellular therapy product manufacturing can be temporarily stored at a refrigerated temperature (i.e., 2–8 °C), based on the results from apheresis material stability studies conducted by each pharmaceutical company prior to approval for manufacturing of therapeutic products (Fig. 1) [3, 9, 10]. This approach minimizes the potential costs of preservation. However, due to geographical constraints and potential logistic challenges, fresh apheresis materials may experience delayed shipment and consequently undergo degradation, which can lead to lower viability upon arrival at the manufacturing site [10]. Conversely, if fresh apheresis materials can be stably cryopreserved before production, the impact of logistics delays can be mitigated. Previous studies have indicated that CAR-T products from cryopreserved apheresis material have comparable in-vitro anti-tumor potency and specificity to those from fresh apheresis material, implying non-inferior clinical outcomes [11]. One retrospective evaluation showed that, even though cryopreserved apheresis material might have reduced cell viability of the starting fraction (peripheral blood mononuclear cells) compared with fresh apheresis material, cryopreservation did not affect final CAR-T fold expansion, transduction efficiency, CD3+%, or CD4+/CD8 + ratios, and also showed no difference in CAR-T persistence and clinical response [12]. Cryopreservation also ensures high stability of starting materials and, in the case of local cryopreservation, also allows leukapheresis to occur when optimal T cells can be harvested (e.g., early in the disease course or before continuous exposure to anti-neoplastic agents) and thereby potentially leads to enhanced CAR-T therapy outcomes [13]. Furthermore, cryopreserved apheresis materials offer several advantages over fresh apheresis materials preserved hypothermically, including post-thaw cell viability, recovery and CD3 + expression [14]. In long-term stability studies to extend shelf life, the post-thaw analysis also suggested comparable viable cell recovery between 30 month- and 6 week-cryopreserved apheresis material [15].

Therefore, cryopreservation of fresh apheresis material via a cryogenic cold chain is considered one of the mitigation approaches to manage logistic and manufacturing capacity challenges as it can prolong the shelf life of cellular starting materials, assure stable quality, and offer scheduling flexibility [10]. Cryopreservation has, therefore, been adopted by many pharmaceutical companies to overcome the challenges of managing manufacturing slots, and de-risking the logistical process and constraints [12].

However, cryopreservation involves initial capital investment, such as for maintenance of a controlled environment and the specific equipment to allow minimum manipulation, if needed. By breaking down the cost of goods, the detailed cost drivers behind the cryopreservation of fresh apheresis materials primarily include facility/utility maintenance and technical support, raw materials, and labor (training, operation) [16]. Cost-effectiveness thereby depends on the level of regulatory requirements applied to those cost drivers. In the case of local cryopreservation, established QMS robustness may vary between medical institutions, including in relation to material procurement, capacity planning, deviation management, and product release. To mitigate this challenge to initial investment for procuring facilities’ capacity and establishing QMS, application of a closed system in a manufacturing process is one of the most effective solutions, as a fit-for-purpose process, to reduce the risk of microbial contamination and improve product safety while reducing initial costs associated with facilities/utility maintenance equipment, and personnel [17, 18].

Conventionally, apheresis formulation cryopreservation was performed in open systems by using bags, tubes, bottles and vials for centrifuge, washing, adding cryopreservation medium and dispensing, which is part of the process of cell collection as the preparation stage for the following CAR-T therapy product manufacturing in academic/clinical cell therapy laboratories. This preparation process is considered as minimal manipulation since it did not alter the properties of human cells and tissues [19]. The open system process is prone to have higher risk of microbial contamination due to lack of physical barriers to the exterior environment, even though air quality can be mitigated by operating in classified cleanrooms. On the other hand, closed systems for the apheresis formulation cryopreservation, i.e. using sterile tubing welder to connect the sterile devices/bags for the sampling and the cell transfer, is a better, more cost-effective and low-risk processing strategy, with less requirement on the classification of the cleanroom, avoiding open contact points and maintaining sterility throughout the process [20, 21]. By employing the closed system in the minimal manipulation such as centrifuging, freezing, thawing, and other processes that do not alter the characteristics and properties of biological cells, not only is contamination from the environment effectively prevented, but costs associated with environmental control measures, gowning, and training are also significantly optimized.

Based on the approach, validated apheresis formulation and cryopreservation processes in the closed system can be executed in a less stringent air classification, such as a controlled, non-classified space. However, this is only subject to minimal manipulation that is clearly described in the U.S. (21CFR1271 HCT/P) [22], EU (EU Annex 1, 1394/2007) [23] and the regulatory framework of certain APAC countries. If there is no clear definition of minimal manipulation within the regulatory framework, the manufacturing process is considered equivalent to substantial manipulation (i.e., more than minimally manipulated). This substantial manipulation includes biological characteristics, function, or viability of human cells and tissue modified during genetic modification and cell culture expansion, which is subject to more stringent regulatory requirements (Current Good Manufacturing Practice, cGMP) at the manufacturing sites when compared to minimal manipulation in the cell therapy laboratories.

In the case of centralized cryopreservation at manufacturing sites, regulatory environments for apheresis formulation and cryopreservation in a closed system fall under the regulations of GMP or GCTP. For example, manufacturing within Japan, an established QMS for formulation and cryopreservation of cellular starting material is required according to GCTP ordinance by the Japanese Ministry of Health, Labor, and Welfare (MHLW).

From an APAC perspective, careful evaluation of logistical, cost, and quality control aspects are crucial for successful manufacturing of CAR-T therapeutic products. Evaluation should also consider the geographic access of certain regions and their impact on cryopreservation logistics. Optimal coverage of all regions is important for access to treatment, which may be challenging in remote or rural areas. Therefore, the quality of fresh apheresis material is impacted by patient access to apheresis sites and a stable logistical plan associated with cryopreservation, which must be carefully considered.

### Cryopreservation Logistics Considerations

1. Logistics and costs.
2. Geography: patient access (patient population, remote area), transit time from an apheresis site to a drug product manufacturing site or cryopreservation site.
3. Stability of fresh cellular starting materials and critical quality attributes.
4. Control of chain of identity and custody (tracking from the donor to the patient and infusion).
5. Transportation method and impact on fresh apheresis material quality during transportation.

### Considerations for Performing Local Cryopreservation (Medical Institutions)

1. Regulations for cryopreservation of cellular starting materials and facility.
2. Establishment/technical transfer of fit-for-purpose processes optimized for reducing quality risk and cost such as application of a closed system.
3. Financial resources and personnel for the initial investment in facility and QMS establishments and continuous operations/improvements (including the insurance reimbursement system).
4. Status of QMS (i.e., accredited QMS including FACT-JACIE and ISO 9001:2015 or ISO 15189:2022 etc.)

### Considerations for Performing Central Cryopreservation (Manufacturing Site)

1. Regulations for cryopreservation of cellular starting materials and facility and timeline to meet the requirements (such as obtainment of GMP license).
2. Establishment/technical transfer of fit-for-purpose processes optimized for reducing quality risk and cost as an application of closed system.
3. Robust QMS, including production capacity management and training and qualification of personnel.
4. Other: Status of regulatory compliance, experience in commercial production etc.

### QMS and Local Regulatory Requirements in APAC

Available regulations in the US (21CFR1271) [22], Europe (EU Annex 1, 1394/2007) [23], and Canada, regard apheresis formulation and cryopreservation as minimal manipulation or equivalent and medical institutions tend to follow FACT/JACIE standards [24], which are audited by the accredited organization and/or should be followed by Good Tissue Practice as per local regulations.

Regulatory compliance requirements for cryopreservation of leukapheresis starting material in APAC countries such as Japan, South Korea, and Australia are shown in Table 1. Relatively few medical institutions in the APAC region are accredited by FACT/JACIE (Table 2). Therefore, apheresis formulation and cryopreservation are still mainly performed by manufacturing sites under country-specific regulations and robust QMS. Consequently, regulation of cryopreserved apheresis materials in the APAC region can be challenging for medical institutions. To tackle this challenge, the regulatory framework for advanced therapy medicinal products (ATMPs) includes a fit-for-purpose risk-based quality framework that minimizes overburdening of regulatory requirements, the importance of which has also been recognized by US Food and Drug Administration (FDA) and European Medicines Agency (EMA) regulatory guidance.

**Table 1:** Applicable regulation for apheresis formulation and cryopreservation for commercial CAR-T cell therapy

Country	Classification	Facility	Category of the practice of autologous cell therapy cryopreservation	Relevant regulatory agency and documentation
Japan	Minimal manipulation	At a medical institution	Medical Practice, Qualification by Marketing approval holder	• Japanese MHLW (“Good gene, cellular and tissue-based products manufacturing practice. 2014.”) ordinance No.93.
		Outside a medical institution	Manipulation before CAR-T production licenced by PMDA (Gene, Cellular, and Tissue-based Products Manufacturing Practice)	
	More than minimal manipulation	Manufacturing sites		
South Korea	Minimal manipulation*	At a medical institution	Medical Practice and licensed by MFDS (Medical Service Act)	• Ministry of Health and Welfare (Act on the Safety of and Support for Advanced Regenerative Medicine and Advanced Biological Products, ARMAB Act) • Ministry of Food and Drug Safety (Regulations on Approval and Safety of Human Cells, etc. and Advanced Biopharmaceuticals)
		Outside a medical institution	Manipulation before CAR-T production licensed by MFDS (Pharmaceutical Affairs Act)	
	More than minimal manipulation	Manufacturing sites		
Australia	Minimal manipulation	At a medical institution	Medical Practice (Australian Regulatory Guidelines for Biologicals)	• TGA (“Australian Regulatory Guidelines for Biologicals [ARGB]). Method of preparation: Interpretation of minimal manipulation.”) • Australian Code of Good Manufacturing Practice for human blood and blood components, human tissues and human cellular therapy products (Australian GMPs for HCT products)
		Outside a medical institution	Manipulation before CAR-T production (Australian Regulatory Guidelines for Biologicals)	
	More than minimal manipulation	Manufacturing sites		

Abbreviations: ARGB, Australian Regulatory Guidelines for Biologicals; ARMAB, Act on the Safety of and Support for Advanced Regenerative Medicine and Advanced Biological Products; CAR-T, Chimeric antigen receptor T-cell; GCTP, Good Gene, Cellular, and Tissue-based Products Manufacturing Practice; GMP, Good Manufacturing Practice; HCT, human cellular therapy; MFDS, Ministry of Food and Drug Safety; MHLW, Ministry of Health, Labor, and Welfare; PMDA, Pharmaceuticals and Medical Devices Agency; TGA, Therapeutic Goods Administration

*Act on the Safety of and Support for Advanced Regenerative Medicine and Advanced Biological Products

Article 2 − 1. “…That excluded from here are operations prescribed by Presidential Decree, which are performed by using simple separation, cleaning, freezing, thawing, or other minimum manipulation of cells or tissues to the extent that their biological properties are maintained.”

Article 2–5. The term “advanced biopharmaceutical” means any of the following drugs defined in subparagraph 4 of Article 2 of the Pharmaceutical Affairs Act: (a) Cell therapy products: Drugs manufactured through physical, chemical, or biological manipulation, such as in-vitro culturing, multiplying, or selecting of living cells from humans or animals. Excluded from here are those prescribed by Ordinance of the Prime Minister, which are manufactured through minimal manipulation, such as simple separation, cleaning, freezing, and thawing to the extent that biological properties are maintained

**Table 2:** Numbers of FACT/JACIE-accredited institutions in the APAC region (current as of May 2025)

Country	FACT (Common for Cellular Therapies, Hematopoietic Cellular Therapy, Cord Blood Banking, Immune Effector Cells)	JACIE
Australia	11	1
Japan	0	0
South Korea	0	0
Singapore	3	1
Hong Kong	2	0
India	2	0
New Zealand	1	0
Taiwan	1	0

Abbreviations: APAC, Asia-Pacific; FACT, Foundation for the Accreditation of Cellular Therapy; JACIE, Joint Accreditation Committee, ISCT and EBMT

### Japan

In Japan, apheresis collection, formulation, and cryopreservation can be performed in medical institutions only after the health authority determines that GCTP regulations do not apply. Consequently, the marketing authorization holder is required to qualify the medical institution as a supplier of the apheresis materials. Since medical institutions in Japan are not under the regulation of GMP/GCTP, and currently there are no FACT/JACIE-accredited clinical sites in Japan, such locations tend to establish a QMS based on guidelines issued by The Japan Society of Transfusion Medicine and Cell Therapy (JSTMCT) and The Japan Society for Hematopoietic Cell Transplantation (JSHCT). These were created based on FACT/JACIE 2006 (3rd Edition Part C and Part D) with an emphasis on quality control, standard operating procedures, and record keeping [25]. Medical institutions in Japan tend to develop QMS following this guideline and ISO 9001:2015 and/or ISO 15189:2022 within medical institutions incorporating the concept of GMP/GCTP. QMS based on ISO 9001 or ISO 15189 are similar to the system required for CAR-T materials and products [26]. However, since these ISO standards were not originally established for blood cell administration or CAR-T practice, the QMS at medical institutions are required to bridge the gaps between these ISO standards and QMS under GMP/GCTP regulations for CAR-T cell therapy. Such gaps include the prevention of contamination of the raw materials and chain of custody and chain of identity management [27].

In the case that a health authority concludes that GCTP regulations are applicable to the formulation and cryopreservation processes of apheresis materials, the facility that will perform the formulation and cryopreservation process of apheresis materials must obtain a license as a GCTP manufacturing site.

### South Korea

In South Korea, the Ministry of Food and Drug Safety (MFDS) has established guidelines (Regulations on Approval and Safety of Human Cells, etc. and Advanced Biopharmaceuticals) for the manufacture of advanced regenerative medicine and biological products, which require cryopreservation to be conducted under a controlled environment [28]. Further, the South Korean government has issued legislation (ARMAB), which specifies procedures such as centrifuging, freezing and thawing as minimal manipulation that are applicable to medical institutions and manufacturing sites [6]. The Act underwent an amendment on August 11, 2020, under Act No. 17472. The amendment streamlined approval process for regenerative medicine clinics and introduced a simplified approval process to facilitate the development and commercialization of advanced regenerative medicine products. The process involves submitting an application for approval to MFDS and obtaining a license.

### Australia

In Australia, the National Pathology Accreditation Advisory Council specifies detailed regulations for the collection, processing, and storage of cellular therapy products, including cryopreservation [29]. These include the need for specific standards in relation to cryopreservation medium and concentration, maximum cell concentration that can be frozen, cooling rate(s), endpoint temperature of cooling, storage temperature and alternative validated procedures in freezing process equipment failure [29]. The Australian Therapeutic Goods Administration (TGA) has also issued guidance (Australian Regulatory Guidelines for Biologicals) in relation to minimal manipulation [19]. In this context, minimal manipulation falls within the regulatory scope of the Australian Code, which is overseen and regulated by TGA. Therefore, formulation and cellular starting materials at a central cryopreservation site can be regulated by Australian GMPs for HCT/Ps depending on the quality attributes and controls [4].

Unlike other APAC countries, large CAR-T cell collection medical centers in Australia tend to follow FACT/JACIE standards and thereby refer to International Society of Blood Transfusion (ISBT) 128, a specific labelling requirement covered under FACT/JACIE standards. Several Australian centers also comply with Good Tissue Practices, Australian GMPs for high throughput cellular phenotyping, and National Association of Testing Authorities certification. Hospitals which are not certified by FACT/JACIE standards are particularly affected in terms of local cryopreservation practice due to differences between the hospitals’ own CAR-T QMS and FACT/JACIE-specific requirements.

### Local Cryopreservation and Central Cryopreservation

In the APAC region, the geographical distance between patients and medical institutions could be a potential challenge when making cryopreservation decisions and strategies. Local cryopreservation hereby refers to freezing fresh cellular starting materials at medical institutions where the materials are collected (i.e. internal cell processing center) at medical institutions for internal and/or affiliated apheresis sites. The cryopreserved materials are then shipped directly to CAR-T manufacturing facilities. Central cryopreservation refers to the cryopreservation of fresh cellular starting materials at cryopreservation sites external to the medical institutions. Here, the materials are collected and shipped from medical institutions to the central Cryopreservation Centers (CPCs), where they are then cryopreserved and finally shipped to CAR-T manufacturing facilities.

### Advantages of Local Cryopreservation in APAC

There are significant advantages of performing cellular starting material cryopreservation locally at medical institutions/local CPCs of APAC regions. Firstly, cryopreservation can begin quickly after collection and transportation and logistical expenses associated with large distances as well as the need for centralized facilities are reduced (Fig. 1). Local cryopreservation also allows local medical institutions to store cellular starting material for a period of time, enables better control of the supply chain, reduces turnaround time for cellular starting material formulation, and allows for more flexibility in scheduling patient treatments [15]. Also, there are certain risk factors for CAR-T cell manufacturing failure, i.e. growth termination or sterility breach, so cryopreservation flexibility might contribute to patients’ treatment strategy prior to apheresis [30]. Furthermore, local cryopreservation can potentially reduce the need and the cost for maintaining and operating central CPC facility/equipment.

### Challenge of Local Cryopreservation in APAC

The accreditation standards or regulatory framework and QMS for local cryopreservation of starting cellular materials at medical institutions are not mature and sufficient in the APAC region, and therefore not harmonized. Especially in Japan, lack of standardization compared to countries in Europe and North America leads to site-specific QMS (e.g., ISO 9001:2015 or ISO 15189:2022) for minimal manipulation processes performed in medical institutions, and thereby requires an audit conducted by an accredited certification body or pharmaceutical companies as part of their supplier qualification and management (Fig. 1).

Another challenge for local cryopreservation is the complexity in the process, particularly when the processes are required to be compliant with processes specified in the quality agreement with marketing approval holders (MAHs) or manufacturers. In most cases, local cryopreservation sites must adapt to different manufacturers’ requirements for cryoprotectants and protocols, creating operational challenges. Based on those facts in APAC, pharmaceutical companies tend to adopt a central cryopreservation strategy in the APAC region. For example, in the case of Japan, CAR-T MAHs such as Kite Pharma for Yescarta and Bristol Meyers Squibb for Abecma and Breyanzi adopted the central cryopreservation strategy or direct shipment of fresh apheresis materials from medical institutions to a manufacturing site while only Novartis applied the local cryopreservation strategy for Kymriah [31]. However, cost effectiveness and financial sustainability of these two strategies should be further investigated later considering the strategies were adopted upon the new drug approvals and might be improved based on the actual product manufacturing data.

### Impact of not Following FACT/JACIE Standards

FACT/JACIE standards provide guidance to the audit and accreditation of apheresis sites for CAR-T cell therapy involved with pharmaceutical companies. However, APAC sites that do not follow FACT/JACIE standards require a more robust supplier audit process. Companies need to evaluate the capability and the QMS of apheresis sites or medical institutions, which can essentially be regarded as suppliers of cellular starting materials. Variations between the requirements by several pharmaceutical companies may burden apheresis sites and increase the complexity involved in establishing appropriate QMS.

### Capacity and Capability Building

As the demand for CAR-T cell therapies increases, there is a need for capacity and capability building through staff training and material management in the APAC region. Training should cover apheresis formulation/cryopreservation techniques, quality control measures, material procurement/inventory management and regulatory compliance requirements, as well as the chain of custody and chain of identity record systems to ensure the safety and the quality of these autologous CAR-T products. By investing in these measures or receiving fiscal support from governmental entities and other organizational sources, local cryopreservation sites can ensure materials meet quality standards while ensuring their operations are efficient and cost effective.

### Potential Solutions for Local Cryopreservation Challenges

Local cryopreservation strategies should prioritize the implementation of a harmonized cryopreservation model to minimize operational complexity, reduce costs and resources, and mitigate quality risks associated with local cryopreservation practices. An adaptive and risk-based approach that encompasses effective processes, techniques, tracking and inventory management, training, and robust quality controls will empower hospitals to leverage their existing workflows, raw materials, infrastructure, and trained personnel. This approach will also facilitate the introduction and leverage of the most effective technologies designed to uphold product quality and safety. Furthermore, it is essential to foster collaborations with non-profit industry associations, such as the Forum for Innovative Regenerative Medicine in Japan [32]. These organizations play a crucial role in establishing social systems, promoting the industrialization of regenerative medicine, and enhancing collaboration among stakeholders including doctors’ associations and pharmaceutical companies involved in this field. They can also serve as a forum to discuss the QMS standards for local cryopreservation. As another solution, engaging with external partners who have extensive experience and expertise in collection from autologous or allogenic donors and cryopreservation in the U.S. and Europe, can offer valuable insights [33].

### Central Cryopreservation in APAC

Central cryopreservation of cellular starting material is associated with various benefits that can contribute to quality and cost control in the APAC region (Fig. 1). Central CPCs can be designed to supply the specified quality of products under established QMS. By adopting the strategy of using central CPCs, manufacturers can reduce the risk of variability in the cryopreservation process and ensure products meet necessary quality standards. Additionally, central CPCs can remove the financial burden associated with local cryopreservation for CAR-T medical institutions, including the costs for infrastructure, equipment, and personnel. Moreover, while centralized CPCs can enhance the quality and consistency of staff training under an established QMS, it can be more challenging in terms of resources within academic institutions [34]. Finally, manufacturers can optimize their logistics networks by establishing strategic partnerships with transportation providers and leveraging technology to improve tracking of shipments.

### Logistical Strategies: Local Versus Central Perspective

Choice of local or central cryopreservation strategies requires consideration of regional-specific regulations, product-specific aspects, and available logistics.

If there is an established QMS at medical centers and the process is applicable to minimal manipulation under local regulations, local cryopreservation is preferable because of the lower risk in logistics and better material quality. Furthermore, cost savings related to transportation between apheresis and cryopreservation sites could mitigate the risks of supply chain control, while shorter lead time from cell collection to cryopreservation could enhance flexibility of cell collection and cryopreservation, with significant improvement in access to cell therapies. Reduction of logistic challenges associated with shipping cryopreserved apheresis material to centralized CAR-T manufacturing sites, which can be particularly difficult in remote or rural areas, is especially relevant in the APAC region given the vast geographic areas with diverse infrastructure. However, as discussed previously, individual local cryopreservation facilities require initial capital investment, as well as capacity and capability building through training.

If there is no well-established QMS, or initial capital investment is not possible at medical institutions, central cryopreservation is preferable due to the stability of supply and consistency in cryopreservation processes, along with better control of capacity at a manufacturing site. However, central cryopreservation requires robust QMS, logistics, and regulatory strategies between the cell collection and cryopreservation site.

Health insurance systems and reimbursement related to cryopreservation processes should also be considered in different countries among APAC when comparing to the established systems in the US. For example, in Japan, the localization process follows a different health insurance system than that in the US. Further, health insurance reimbursement differs between apheresis and the combination of apheresis and cryopreservation at hospitals in Japan. In Australia, CAR-T therapy is managed as a technology rather than a pharmaceutical product, so funding is sought through the Medicines Services Advisory Committee (MSAC) rather than the Pharmaceutical Benefits Advisory Committee (PBAC). Specific CAR T-cell therapies, such as Kymriah for eligible patients with relapsed or refractory diffuse large B-cell lymphoma, transformed follicular lymphoma and primary mediastinal B-cell lymphoma are manufactured and reimbursed in Australia. This reimbursement covers both the CAR-T product and the relevant manipulation processes involved. The National Health Insurance of Korea also heavily subsidizes certain CAR-T therapies through designated centers approved by the government for handling human cellular starting materials.

Ultimately, individual countries need to consider their unique characteristics related to health coverage, geographic and related transportation constraints, and hospital-based resources to formulate an optimal model for both operation and delivery, and an accompanying fit-for-purpose regulatory framework for cellular starting materials of CAR-T therapies.

## Discussion

CAR-T therapies have led to a reinvention of the supply chain due to the individualized nature of the products, with medical institutions now responsible for supplying cellular starting materials and administering the final product to patients [35]. To ensure the quality and safety of CAR-T products, regulatory frameworks have evolved to allow manufacturers to apply a risk-based approach to control the supply chain of starting materials. With multiple CAR-T therapies on the market and many more in late-stage development, autologous cellular therapies will continue to evolve and the practical logistics around optimal delivery will develop in parallel and become more mature. The uniqueness of this approach demands a regulatory framework that is fit for purpose, balancing the need for timely administration, control measures, and consideration of cost-effectiveness. Practically speaking, it is important to discuss and have cryopreservation strategies aligned before regulatory submission in collaboration with experts in regulatory affairs and quality, market access, cryopreservation techniques of human blood cells and logistics. The health insurance system of the country to reimburse the cryopreservation-related costs should also be carefully investigated and considered. The logistics implemented in clinical trials of CAR-T suggest that, after leukapheresis, fresh materials can be shipped for central manufacturing involving T cell enrichment in a closed system [36]. In another model, material can be formulated and cryopreserved at either local or central sites depending on the regulatory framework required for cellular starting materials [10, 11]. It is essential to develop a cross-functional expert logistics strategy and apply a risk-based approach towards cell starting materials for CAR-T therapy focused on mitigating the overall risk to product quality and safety, based on a deep understanding of the local regulatory framework. In addition, continuous monitoring and improvement should be implemented with a focus on operational efficiency, financial sustainability, and product quality. This can be achieved by employing a risk-based, fit-for-purpose, and adaptive approach. Such systematic evaluation may necessitate adjustments in strategy, which could include transitioning from/to a local to/from a central cryopreservation strategy, shifting to direct shipment of fresh apheresis materials to the manufacturing site, or making other changes tailored to the identified needs and circumstances within the regulatory framework. If successful, this approach should lead to a high-quality CAR-T product with reduced vein-to-vein times, lower cost of goods, and a fit-for-purpose regulatory framework.

## Data Availability

No datasets were generated or analysed during the current study.
